# Effectiveness of bandage contact lens application in corneal epithelialization and pain alleviation following corneal transplantation; prospective, randomized clinical trial

**DOI:** 10.1186/s12886-016-0346-6

**Published:** 2016-10-06

**Authors:** Jun Shimazaki, Chika Shigeyasu, Yumiko Saijo-Ban, Murat Dogru, Seika Den

**Affiliations:** Department of Ophthalmology, Tokyo Dental College, Ichikawa General Hospital, 5-11-13 Sugano, Ichikawa, Chiba 272-8513 Japan

**Keywords:** Corneal transplantation, Bandage contact lens, Corneal epithelium, Pain

## Abstract

**Background:**

To assess the efficacy of bandage contact lens (BCL) application to promote epithelialization and alleviate pain following corneal transplantation.

**Methods:**

Twenty-six consecutive patients who underwent corneal transplantation were randomly assigned to undergo BCL application (BCL group, *n* = 14) or no BCL application (control group, *n* = 12) at the end of the surgery. Corneal epithelialization was analyzed by photography using fluorescein staining, and ocular pain was measured using a visual analog scale.

**Results:**

The mean size of the epithelial defect relative to the graft area in the BCL group was 21.80 ± 35.10 % at the end of surgery, 18.20 ± 31.10 % on postoperative day 1, and 5.45 ± 11.10 % on postoperative days 3 to 5. These values in the control group were 9.64 ± 17.60 % at the end of surgery, 11.50 ± 25.70 % on postoperative day 1, and 0.00 ± 0.00 % on postoperative days 3 to 5. There were no significant differences in the speed of epithelialization between the two groups. The mean preoperative pain score in the BCL group was slightly higher than that control (4.27 ± 1.96 vs. 2.41 ± 2.18, respectively; *P* = 0.039). The scores significantly increased on postoperative day 1 and promptly returned to preoperative levels by day 7 in both groups, and there were no significant differences between the groups.

**Conclusions:**

No significant benefits of BCL application at the time of corneal transplantation were observed in this study. The efficacy and safety of BCLs in eyes with compromised epithelialization require further study.

**Trial registration number:**

UMIN 000019091. Date of registration: 2015/09/22

## Background

Proper postoperative management is a key factor for successful outcomes of corneal transplantation. In particular, prompt epithelialization is essential for avoiding vision-threatening complications such as infection, scarring, and stromal melting [1]. Application of a bandage contact lens (BCL) is one management technique for promoting corneal epithelialization. The use of a BCL was first reported by Gasset and Kaufman in 1970 [2], and BCLs have since been used for pain relief and protection in patients with bullous keratopathy, followed by other indications such as sterile corneal ulcer and keratoconjunctivitis sicca [3, 4]. Application of a BCL following keratoplasty was first reported in the 1970s to promote epithelialization, tamponade of small wound leaks, relief of suture irritation, and smoothing of wound margin irregularities [5, 6]. Improvements in BCL material and technology have enabled the use of high-water-content BCLs for extended wear [7, 8].

Despite wide recognition of the usefulness of BCLs for the postoperative management of corneal transplantation, no detailed studies of this technique have been reported. Therefore, we conducted a prospective randomized study to assess the speed of epithelialization and suppression of postoperative pain associated with BCL application following corneal transplantation.

## Methods

### Study design

In this randomized, prospective, single-center clinical trial, we evaluated consecutive patients undergoing corneal transplantation (penetrating keratoplasty [PKP] or deep anterior lamellar keratoplasty [DALK]) at Tokyo Dental College Ichikawa General Hospital, a referral hospital for corneal transplantation, from October 2006 to May 2008. The protocol adhered to the principles of the Declaration of Helsinki. The manuscript reporting adheres to the CONSORT guidelines for the reporting of randomized trials. Eyes with severe lid/blink abnormalities, total limbal deficiency, and severe dry eye with decreased reflex tearing were excluded. Patients who had diabetes mellitus, those undergone endothelial keratoplasty or combined intraocular surgeries other than cataract extraction were also excluded. The patients were randomly allocated into one of two groups: those who underwent BCL application at the end of surgery (BCL group) and those who did not (control group).

### Surgical procedure and postoperative treatment

All of the eyes underwent corneal transplantation under retrobulbar anesthesia with standardized surgical techniques. The donor corneas were preserved in Optisol-GS corneal storage medium (Bausch & Lomb Inc., Rochester, NY) and were used for corneal transplantation in < 7 days. The grafts were trephinated with a 7.75 mm diameter Barron donor punch (Jedmed Instrument Co., St. Louis, MO), and recipient corneas were trephinated with a 7.50 mm diameter Hessburg-Barron vacuum trephine. The donor grafts were secured by 10–0 nylon sutures with a 24-bite running suture pattern, or a 16-bite interrupted suture pattern. Suture knots were buried. All surgeries were performed by a single experienced surgeon (J.S.).

A Breath-O® BCL (Toray Industries Inc., Tokyo, Japan), a hydrophilic lens comprising vinyl pyrrolidone and methyl methacrylate polymer with 78 % water content, was applied to patients in the BCL group. The lens parameters were as follows: power plano; base curve, 9.0 mm; diameter, 13.5 mm; central thickness, 0.22 mm; and oxygen transmissibility (Dk/L) value, 48 × 10^−11^ (cm^2^/s) [mLO_2_/(mL•hPa)] (SI). The Japanese Ministry of Health, Labour, and Welfare approved this contact lens for continuous use for up to 1 month. BCLs with same parameters were used in each case.

The eye was patched until the first postoperative day. All eyes received following standardized postoperative treatments. They included topical 0.1 % betamethasone (Rinbeta PF®; Nitten Pharmaceutical Co., Nagoya, Japan) and 0.5 % levofloxacin (Cravit®; Santen Pharmaceutical Co., Osaka, Japan) 5 times a day starting 1 day postoperatively. No other eye drops were used until epithelialization was complete. The patients were hospitalized until complete epithelialization had been achieved (at least 7 days). They were examined every day using slit-lamp biomicroscopy and fluorescein staining at approximately the same time of day (around 9:00 a.m.), approximately 1 h later when last eye drops were instilled.

### Analysis of pain

A visual analogue scale (VAS) was used to assess the patients’ pain. The patients responded to a subjective pain scale before surgery and on postoperative days 1, 3 to 5, and 7 following corneal transplantation. The VAS is a 10 cm straight line, the ends of which are defined as the extreme limits of the sensation or response to be measured. The patients marked the position on the scale that indicated their level of ocular pain; the scale ranged from no pain (score of 0) to worst pain imaginable (score of 100) [9].

### Analysis of epithelial defects

The size of the corneal defects was recorded with a cobalt blue filter following instillation of 2 μL 1 % fluorescein solution. The recorded images were analyzed by ImageJ® software (NIH, Bethesda, MD) using the binary process. The size of the epithelial defect was calculated and expressed as that relative to the whole graft area. The examination was performed at the end of the surgery and video-recorded under the operating microscope. The epithelial status was also examined with slit-lamp biomicroscopy on postoperative days 1, 3 to 5, and 7 until total graft healing was achieved. The BCL was removed when epithelialization was complete.

### Examinations

A Snellen chart was used for visual acuity testing, and decimal values were converted into their logarithm for statistical analysis. The Schirmer test without topical anesthesia was performed to evaluate tear secretion. A standardized strip of filter paper (Alcon Inc., Fort Worth, TX) was placed in the lateral one-third of the lower lid, and was left in place for 5 min with the eyes closed. Readings were recorded in millimeters of wetted paper. Any adverse events possibly related to the use of the BCL were recorded.

### Statistical analysis

GraphPad Prism 6.0 software (GraphPad Software Inc., San Diego, CA) was used for statistical analysis. The Mann-Whitney *U* test and Student’s *t*-test were used to compare nonparametric and parametric values, respectively, between the two groups. The chi-squared test with Fisher’s exact test was used for contingence data analysis. A *P* value < 0.05 was considered statistically significant.

## Results

### Patients’ characteristics

In total, 26 eyes of 26 patients were analyzed. There were 14 patients (8 males, 6 females) in the BCL group and 12 patients (5 males, 7 females) in the control group. The demographic data of the two groups are shown in Table 1. In the BCL group, causative diseases included bullous keratopathy (*n* = 7) including one eye with Fuchs’ dystrophy, regrafting (*n* = 3), corneal scarring secondary to interstitial keratitis (*n* = 2), and keratoconus (*n* = 2). One eye with keratoconus underwent DALK, and the other 13 eyes underwent PKP. Seven eyes had combined cataract surgery or intraocular lens insertion, and four eyes had a history of glaucoma. In the control group, 11 eyes underwent PKP, and 1 eye underwent DALK. Causative diseases for corneal transplantation included regrafting (*n* = 5, including one patient who underwent DALK for lattice dystrophy), bullous keratopathy (*n* = 4), and herpes keratitis (*n* = 2). Two eyes underwent combined cataract surgery, and one other eye had a history of glaucoma. The distribution of causative diseases in the two groups did not significantly differ (*P* = 0.70). There were also no significant differences in preoperative visual acuity, intraocular pressure, or Schirmer test values between the two groups (Table 1).

**Table 1:** Demographic data in the bandage contact lens (BCL) and control groups

		BCL	Control	*P* value
		n = 14	n = 12	
Age (years)		64.6 ± 15.9	59.1 ± 15.0	0.69
Male: Female		8:6	5:7	0.70
Preoperative visual acuity (log)		-1.40 ± 0.91	-1.85 ± 1.36	0.33
Number of previous KPs				
0		11	7	0.99
1		3	4	
2		0	1	
Preoperative Schirmer value (mm per 5 min)		9.71 ± 5.76	12.9 ± 11.7	0.38
Surgery	PKP	13	11	1.0
	DALK	1	1	
Diseases	BK	7	4	0.70
	Regraft	3	5	
	Herpes	0	2	
	Scarring	2	0	
	Keratoconus	2	0	

Data are expressed as mean ± standard deviation

*KPs* keratoplasties, *PKP* penetrating keratoplasty, *DALK* deep anterior lamellar keratoplasty, *BK* bullous keratopathy

### Effects on pain relief

**Fig. 1:**
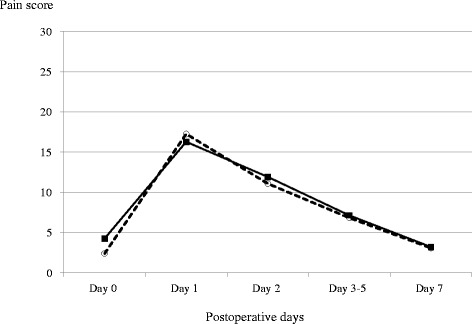
Changes in pain scores assessed using the visual analog scale. BCL group: solid line with square marks; control group: dotted line with circles

Changes in the pain scores in both groups are demonstrated in Fig. 1. The preoperative pain score assessed using VAS analysis was higher in the BCL group than in the control group (4.27 ± 1.96 and 2.41 ± 2.18, respectively; *P* = 0.039). Both of the groups showed significant increases in pain scores 1 day postoperatively (16.3 ± 15.2, *P* = 0.018 and 17.3 ± 13.0, *P* = 0.0021 in the BCL and control groups, respectively). However, there were no significant differences in the postoperative pain scores between the two groups throughout the study period (*P* > 0.05). The scores returned to the preoperative levels by approximately 1 week postoperatively in both groups.

### Effects on epithelial healing

**Fig. 2:**
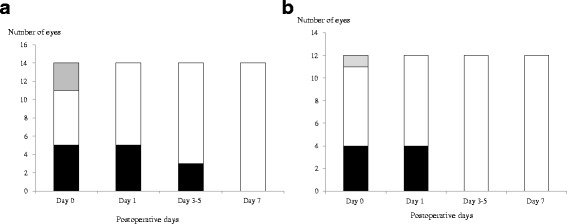
Numbers of eyes with epithelial defects in the (**a**) BCL and (**b**) control groups. Black column: with epithelial defects; white column: without epithelial defects; gray column: data not available

**Fig. 3:**
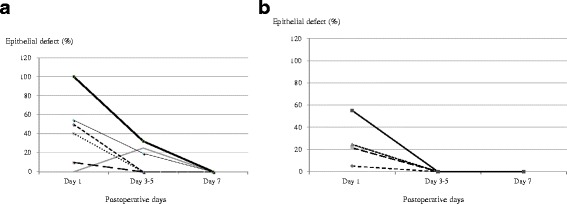
Percent area of corneal defects relative to graft area in (**a**) BCL and (**b**) control groups. Each line represents different eyes

Epithelial defects in the donor grafts were noted in five and four eyes in the BCL and control groups, respectively. On postoperative day 1, five eyes in the BCL group had epithelial defects with a mean percentage area relative to the whole graft area of 18.2 ± 31.1 % (range, 0.0–100.0 %). Four eyes in the control group had epithelial defects with a mean percentage area of 11.5 ± 25.7 % (range, 0.0–71.0 %). The difference between the two groups was not statistically significant (*P* = 0.56). While three eyes in the BCL group exhibited epithelial defects on days 3 to 5, no eyes in the control group exhibited defects (*P* = 0.10). All of the epithelial defects were completely epithelialized within 5 and 7 days in the control and BCL groups, respectively (Fig. 2). The BCLs were discontinued at a mean of 4.3 days after surgery (range, 2.0–7.0 days). The speed of epithelialization in eyes with epithelial defects in the control and BCL groups was not significantly different (Fig. 3).

### Adverse events

The BCLs were well tolerated by all of the patients. No recurrence of epithelial problems was noted following BCL removal. The BCL was spontaneously dislodged on postoperative day 3 in one patient; however, epithelialization was complete on that day, and the BCL was therefore removed. No other complications were noted, such as infection, BCL damage, or corneal infiltration.

## Discussion

Pain management is important following ocular surgeries, including corneal transplantation. Postoperative pain may be caused by various factors such as inflammation, ocular surface irregularities, exposed suture material, and damaged nerve endings. Pain management includes the use of acetaminophen, oral nonsteroidal anti-inflammatories, narcotics, pressure patching, and BCL application. In addition to pain relief, BCLs are used after corneal transplantation for mechanical protection of denuded corneal epithelium, for maintenance of corneal hydration, as a barrier to the eyelids or lashes, for retention of medication, and for general promotion of ocular healing [1]. We used high-water-content soft BCLs in this study, mainly because these lenses are approved for extended wear.

The severity of postoperative pain differs considerably from one patient to another. Postoperative pain following corneal transplantation was reported to be mild by Segev et al. [10], who used a 4-point verbal pain scale. In their study, patients reported a mean score of 0.27 on postoperative day 1, corresponding to an approximate score of 8.9 when converted into a full scoring system of 100. Our results showed slightly higher scores, although they fell within the same mild pain category. We found no significant differences in the pain score between eyes with and without BCLs. Patients in both groups experienced transient increases in postoperative pain followed by a prompt return to preoperative levels (Fig. 1). Because the corneas are in a denervated condition following corneal transplantation, the pain may be mainly caused by damaged nerve endings, and not by surface irregularities. Therefore, it is plausible to consider that the application of BCLs has limited value because its usefulness seems to be exerted through protection of surface irregularities.

Obtaining stable ocular surface epithelialization is the first step for success in corneal transplantation. However, several factors interfere with the achievement of this goal, including donor- and surgery-related problems such as a poor donor epithelial status, long preservation time, and large trephination size [11–14]. Recipient-related factors include lid problems, decreased tear secretion, poor limbal function, diabetes mellitus, and the use of various topical medications that may affect the epithelialization [9, 15–17]. In eyes without wound healing problems, epithelialization is completed within a median period of 2 days following corneal transplantation with wide variation [15].

Various approaches to the treatment of epithelial problems have been reported. Treatment usually starts with mild intervention including pressure patching, topical lubricants, ointment, and punctual occlusion [11, 14, 16]. Reevaluation of topical medications is also important (e.g., those containing preservatives such as benzalkonium chloride may be best to omit from the treatment regimen). If epithelial defects persist, more aggressive approaches such as tarsorrhaphy or amniotic membrane patching should be considered. The application of BCLs is an alternative approach to promote epithelialization. BCLs provide protection usually provided by the lids; this protection is needed to allow migrating epithelial cells to develop proper adherence to the underlying basement membrane, promoting epithelialization [18]. However, it has also been reported that the prolonged use of BCLs may be associated with a risk of infectious keratitis [19].

In this study, no significant differences were found in the epithelialization speed between eyes with and without BCLs. One of the reasons for the absence of this effect may be the relatively healthy donor corneal epithelium; 6 of 14 eyes had no epithelial defects at the end of surgery. Many of the eyes with epithelial defects showed mild to moderately sized defects (up to approximately 50 %) 1 day postoperatively. In another study, 50 of 66 grafts (75.8 %) showed epithelial defects at the end of corneal transplantation with a median defect size of 20 % [15]. Our results appear to be better than those in this previous report, presumably because of the difference in the storage medium used (McCarey-Kaufman medium vs. Optisol GS). The effects of BCLs on epithelialization may be obscured when the donor corneal epithelium is in good condition. Another explanation may be that our patients had relatively normal tear secretion, blink/lid function, and limbal function. Wearing BCLs may be advantageous on a more compromised ocular surface. Further studies in patients at high risk for poor epithelialization are needed to clarify this point. In addition, the epithelial surface seemed to be smoother in the BCL groups than in the control group. The epithelial integrity may be better with the presence of a BCL. More detailed analysis involving measurement of the epithelial permeability or confocal microscopic examination may be necessary.

There were some drawbacks in the present study, such as the small sample size. It is well known that the fitting of BCL is influenced by many factors such as condition of donor/recipient corneas, surgical technique, and postoperative treatments. In order to minimize the bias, we only included cases performed by a single surgeon who used standardized surgical technique. We also applied the same postoperative treatments. However, it should be noted that there were some differences in causative diseases for corneal transplantation between the BCL and control groups, although the difference in disease distribution did not significantly differ. Also, in this prospective study, we included both eyes with and without epithelial defects at the end of surgery. Advantages of BCL on epithelial healing may become evident when applied only to the corneas with damaged epithelium. The other drawback was that we used high water content hydrogel bandage lenses as BCL because they were the only one that was approved at the time of initiation of the study. Recently, higher Dk silicone hydrogel bandage lenses have been replacing the conventional soft contact lenses. The use of more biocompatible silicone hydrogel-based BCL might lead a positive impact on the results. We are planning to conduct another study to examine the efficacy of silicone hydrogel contact lenses on postkeratoplasty eyes.

## Conclusions

The present study failed to show advantages of BCLs on suppression of postoperative pain or promotion of epithelialization following corneal transplantation. Considering the cost and additional management associated with the use of BCLs, we do not recommend using BCLs in all cases. BCLs may have advantages when used in eyes at risk for prolonged epithelialization.

## Abbreviations

BCL: Bandage contact lens
DALK: Deep anterior lamellar keratoplasty
PKP: Penetrating keratoplasty
VAS: Visual analogue scale
